# Nutrient Adequacy in Endurance Athletes

**DOI:** 10.3390/ijerph20085469

**Published:** 2023-04-11

**Authors:** Kamiah Moss, Andreas Kreutzer, Austin J. Graybeal, Yan Zhang, Robyn Braun-Trocchio, Ryan R. Porter, Meena Shah

**Affiliations:** 1 Department of Kinesiology, Texas Christian University, Fort Worth, TX 76129, USA; 2 Physical Medicine and Rehabilitation, Baylor Institute for Rehabilitation, Dallas, TX 75246, USA; 3 School of Health Promotion & Kinesiology, Texas Woman’s University, Denton, TX 76204, USA; 4 School of Kinesiology & Nutrition, College of Education and Human Sciences, University of Southern Mississippi, Hattiesburg, MS 39406, USA; 5 Harris College of Nursing & Health Sciences, Texas Christian University, Fort Worth, TX 76129, USA

**Keywords:** nutrient intakes, endurance athletes, nutrient adequacy macronutrients, micronutrients

## Abstract

Proper nutrition is critical for optimal performance in endurance athletes. However, it is unclear if endurance athletes are meeting all their energy and nutrient needs. We examined if endurance athletes are meeting their nutritional requirements and if this differed by sex. Ninety-five endurance athletes (n = 95; 50.5% men; 34.9 ± 12.9 y) participated in the study. Dietary intake was evaluated using the 24 h dietary recall method. Energy and nutrient intakes were calculated using the ESHA Food Processor Diet Analysis Software and compared against reference nutrient intakes. Endurance athletes did not consume the recommended amount of energy (76.8% of athletes), carbohydrates (95.8%), linoleic acid (75.8%), α-linolenic acid (ALA) (77.9%), eicosatetraenoic and docosahexaenoic acid (96.8%), dietary fiber (49.5%), vitamins D (93.7%), E (71.6%), and K (54.7%), folate (54.7%), pantothenic acid (70.5%), biotin (83.2%), manganese (58.9%), magnesium (56.8%), chromium (91.6%), molybdenum (93.7%), choline (85.3%), and potassium (56.8%), and consumed too much saturated fat (50.5%) and sodium (94.7%) than recommended. Fisher’s Exact test showed that the requirements for dietary fiber (70.8% vs. 27.7%), ALA (87.5% vs. 68.1%), and total water (70.8% vs. 44.7%) were not met by more men versus women (*p* < 0.05). The needs for protein (70.2% vs. 25%) and vitamin B12 (46.8% vs. 22.9%) were not met by more women compared to men (*p* < 0.05). These findings need to be confirmed by a larger study.

## 1. Introduction

Nutrition is one of the key factors in achieving optimal training, performance, recovery, injury prevention, and health among endurance athletes [1,2]. More specifically, endurance athletes train at high volumes which may increase susceptibility to fatigue and muscle damage, negatively impacting performance [3]. To mitigate the effects of prolonged or rigorous training, athletes rely on nutrition to aid in recovery and maintaining health [4]. Studies have also shown that prolonged inadequate energy and nutrient intakes can lead to increased risk for overtraining syndrome (i.e., when training exceeds the ability to recover), infectious diseases, and stress fractures [5,6,7]. Other health issues related to prolonged inadequate energy balance or relative energy deficiency in sport include complications impacting the gastrointestinal, endocrine, reproductive, skeletal, renal, cardiovascular, and central nervous systems [8]. Despite the importance of proper nutrition, previous studies have shown that many athletes do not achieve adequate energy, macronutrient, and micronutrient intakes [9].

Cross-sectional studies have consistently found that a high percentage of athletes do not meet the recommendations for energy and some macronutrient intakes. Inadequate energy intake has been found in U.S. female collegiate cross-country and lacrosse athletes [10,11], elite Brazilian athletes [12], and South Asian athletes [13]. In addition, several studies from different countries have shown that athletes do not get enough carbohydrates [9,14,15,16,17], protein [1,18], dietary fiber, and essential fatty acids such as linoleic acid, alpha-linolenic acid (ALA), eicosapentaenoic acid (EPA), and docosahexaenoic acid (DHA) [6,16]. Moreover, female athletes are less likely to meet the recommended amount of dietary fiber, possibly because they consume fewer calories than men [16]. In contrast to these findings multiple studies have found that many athletes consume more total fat than the recommended amount (20–35% energy) [15,16,19]. Besides inadequate consumption of several macronutrients, many athletes have reported inadequate intakes of thiamin, riboflavin, niacin, vitamin B_6_, vitamin B_12_, pantothenic acid, biotin, folate, vitamins A, C, D, E, and K, potassium, calcium, zinc, phosphorus, manganese, chromium, molybdenum, selenium, and magnesium [9,16,18,19,20,21,22,23,24,25,26]. Further, female athletes are less likely to meet the recommendations for iron intake compared to male athletes [9,16,18,20,27] and female athletes may have lower intakes of vitamin B_12_ [28].

Although inadequate nutrient intakes and the impact of this on athletes have been noted, there are several limitations in the current literature. For example, a majority of the studies that examined macronutrient and micronutrient intakes were conducted in elite athletes from a mix of various types of sports [6,9,11,12,15,18,20,21,22,26]. The few studies that examined nutrient intakes in endurance athletes only, primarily focused on macronutrients [10,17]. and a few micronutrients [16,19,23]. Additionally, there were even fewer studies in endurance athletes examining essential fatty acids such as linoleic acid and ALA [16]. Further, examining essential fatty acids intakes in endurance athletes is critical because they aid in body weight maintenance and reduce inflammation [29,30,31].

To address gaps in the previous literature, the primary aim of this study was to determine if endurance athletes are meeting their requirements for a comprehensive set of macronutrients including essential fatty acids and micronutrient intakes. Capturing this information is of critical importance given that adequate nourishment is needed for optimizing training, performance, injury prevention, injury recovery, and health among endurance athletes [4]. The secondary aim of this study was to compare nutrient adequacy by sex. Based on previous literature, we hypothesized that the majority of endurance athletes will not meet the nutrient requirements and that fewer female athletes will meet the recommendations compared to male athletes.

## 2. Materials and Methods

### 2.1. Participants

A total of 95 (women: 47, men: 48) endurance athletes were recruited by emailing fliers to endurance sports clubs and linking them to their social media groups, posting fliers on campus and sport stores in the Dallas–Fort Worth metroplex, and by word of mouth. The endurance athletes were Tier 2: trained/developmental or above based on the criteria from McKay and colleagues [32]. The criteria for Tier 2 training involves training at the local level, exercising ≥ 3 days per week, identify with a specific sport, train with the purpose to complete, have limited skill or development [32]. About 80% of the athletes lived in Texas and the remainder were from other states within the U.S. Inclusion criteria included individuals competing in and/or training for recreational competitive endurance-based sports (e.g., cycling, running, rowing, triathlon, and/or swimming). The exclusion criteria included individuals under 18 years old and those who did not self-identify as an endurance athlete or participate in training activities for endurance-based sports. 

This study was approved by the Institutional Review Board and each participant read and signed an approved informed consent form. Data collection began in January 2021 and ended in May 2022.

### 2.2. Procedures

Athletes who were eligible to participate were sent an electronic informed consent document which they signed and returned to the research team via email. Following this, a phone interview was scheduled with each participant to collect information on demographics, anthropometry, dietary intake, health information, and medication and supplement use. Each member of the research team was trained by one of the authors (KM) on how to collect the study data prior to conducting the interviews.

### 2.3. Measurements

#### 2.3.1. Demographics, Anthropometry, Type of Sport, and Health History

Demographic, anthropometric, type of sport, and health history were collected via questionnaire. Demographic information included age, sex (male and female), ethnicity (Hispanic and non-Hispanic), race (White and other including Black/African American, Asian, American Indian/Alaska Native, Native Hawaiian/Other Pacific Islander, and multiracial), and education (high school diploma or lower, some college, college degree, and graduate degree or higher). Self-reported height (m) and weight (kg) were used to calculate body mass index (BMI) (kg/m^2^). Type of sport included categories such as cycling, running, rowing, swimming, and triathlon. Health history included questions regarding the participant having any chronic health condition. Additionally, the participants were asked if they used any medications, supplements, were vegetarian, currently smoked, or consumed alcohol. 

#### 2.3.2. Dietary Intake Assessment

Dietary recalls were collected using a multiple-pass 24 h dietary recall method, a validated measure [33,34]. The 24 h dietary recall was collected only after first verifying with the participant that the intake on the previous day was representative of their usual intake. The interview was re-scheduled for another day if the intake on the previous day was reported by the participant not to be similar to their usual intake. 

During the multiple-pass 24 h recall, participants were asked to remember and report, in detail, the type and amount of all the foods and beverages that they had consumed on the previous day. The multiple-pass 24 h recall method consisted of five steps [34]. In the first step, the participants provided a list of all of the foods and beverages they consumed. During the second step, participants were asked if they consumed any foods from the commonly forgotten list such as beverages, snacks, fruits, vegetables, and breads. In the third step, participants were asked about the time and occasion they consumed the foods. During the fourth step, participants provided details about the amounts of food they consumed. Participants were asked to determine portion sizes using tablespoons, teaspoons, cups, pounds, ounces, grams, or slices. The participants were encouraged to provide brand names for processed food, restaurant names if they ate out, and recipes for the food they prepared at home. In the fifth step, the researcher asked additional questions about how the food was prepared and foods that the participant might have missed such as snacks or beverages. 

Energy and nutrient intake were determined using ESHA’s Food Processor Diet Analysis Software Version 11.11 (Salem, OR, USA). The ESHA’s Food Processor Diet Analysis software provides detailed reports on macronutrient and micronutrient intakes. The software database consisted of over 1900 food sources such as the USDA Standard Reference database, USDA FoodData Central Brands, and manufacturer’s data. Further, the software database has more than 146,000 ingredients, recipes, and restaurant food brands.

#### 2.3.3. Dietary Nutritional Adequacy

The dietary nutritional adequacy was determined by calculating the percentages of participants who had nutrient intakes below the recommended standards set by the by the Institute of Medicine (IOM) [35], American College of Sports Medicine (ACSM) [36], the Dietary Guidelines for Americans (DGA) [37], or the American Heart Association (AHA) [38]. The ACSM recommendations for athletes were used for assessing dietary fat, protein, and carbohydrate adequacy [36]. Estimated Average Requirements (EAR) a Dietary Reference Intake (DRI), set by the IOM, and used to assess the nutrient adequacy of a group of individuals, were used to assess adequacy for omega 3 fatty acid, omega 6 fatty acid, vitamins A, C, D, E, B_6_, and B_12_, thiamin, riboflavin, niacin, folate, calcium, copper, iron, magnesium, phosphorus, selenium, molybdenum, and zinc [39,40,41,42,43,44]. Adequate intake (AI), another DRI, was used for the nutrients that did not have an EAR to determine the number of participants who had inadequate intakes of linoleic acid, ALA, total dietary fiber, vitamin K, pantothenic acid, biotin, manganese, chromium, choline, potassium, sodium, and total water [35,40,42,45]. Estimated energy requirement (EER), a DRI, was used to determine energy needs [46]. Percent energy from saturated fat was assessed using the 2020–2025 DGA [37]. The AHA guidelines were used to determine the number of participants who did not meet the recommendations for EPA, DHA, and dietary cholesterol [38].

### 2.4. Statistical Analysis

Categorical variables (i.e., race, ethnicity, education, endurance sport, smoking status, alcohol consumption, vegetarian status, chronic conditions, medication use, and supplement use) were presented as percentages and continuous variables (age and BMI) as mean ± standard deviation. Energy, macronutrient, and micronutrient intakes were presented in medians (25th and 75th percentiles) due to skewed distributions. Differences by sex for categorical variables were determined using Fisher’s Exact test and continuous variables by Wilcoxon Rank-Sum test. Since nutrient variables had skewed distributions, the Wilcoxon Rank-Sum tests were used to compare energy, macronutrient, and micronutrient intakes by sex. 

Nutrient adequacy was determined by calculating the proportion of athletes with inadequate nutrient intakes in comparison to the reference values. Fisher’s Exact test was used to compare the proportion of female and male endurance athletes that did not meet the energy, macronutrient and micronutrient requirements. Similar comparisons were made by type of sport and whether or not they had a health condition, and no differences were found. These data were not reported in the paper. Data were analyzed using IBM SPSS version 29 (Armonk, NY, USA). Alpha level was set at 0.05.

## 3. Results

### 3.1. Participant Characteristics

Participant characteristics for the total sample are presented in Table 1. Mean ± standard deviation for age and BMI were 34.9 ± 12.9 y and 23.6 ± 3.84 kg/m^2^, respectively. Slightly more than half (50.5%) of the participants were male, 91.6% were white, 83.2% were non-Hispanic, 71.5% had a bachelor’s or graduate degree, 49.5% were runners, 24.2% were triathletes, 18.9% were cyclists, 5.3% were rowers, and 2.1% were swimmers. None of the participants smoked, 66.3% consumed alcohol, 7.4% were vegetarian, 64.2% had a chronic condition, 36.8% were on medications, and 49.5% used supplements. None of the participant characteristics were different by sex.

### 3.2. Energy and Macronutrient Intakes

Energy and dietary macronutrient intakes are presented in Table 2. Male endurance athletes consumed significantly more energy (median difference (MD): 541 kcal/d; *p* ≤ 0.0001), and dietary cholesterol (MD: 152 mg/d; *p* < 0.01) compared to female endurance athletes. 

### 3.3. Micronutrient Intakes

Micronutrient intakes are presented in Table 3. Female endurance athletes consumed significantly less vitamin B_12_ (MD: 1.9 μg/d; *p* ≤ 0.001), iron (MD: 5.3 mg/d; *p* < 0.01), selenium (MD: 26.7 μg/d; *p* = 0.02), choline (MD: 87 mg/d; *p* = 0.03), and zinc (MD: 2.6 mg/d; *p* = 0.03) compared to male endurance athletes. 

### 3.4. Proportion of Participants with Inadequate Nutrient Intakes

The proportion of participants that did not meet the energy, macronutrient, and micronutrient requirements is presented in Table 4. More than 50% of male athletes did not consume enough energy, carbohydrates, linoleic acid, alpha-linolenic acid, EPA and DHA, dietary fiber, vitamins D, E, and K, pantothenic acid, biotin, manganese, magnesium, chromium, molybdenum, choline, potassium, and total water, and another 30–50% had insufficient vitamins A and C, thiamine, folate, calcium, and zinc intakes. Among women, >50% did not consume enough energy, protein, carbohydrates, linoleic acid, alpha-linolenic acid, EPA and DHA, vitamins D, E, and K, folate, pantothenic acid, biotin, manganese, chromium, molybdenum, choline, and potassium, and 30–50% had insufficient vitamin A, thiamine, vitamin B_12,_ calcium, magnesium, selenium, zinc, and total water. About 40–56% of the male and female athletes consumed more than the recommended amount of cholesterol and total and saturated fat, and more than 90% consumed too much sodium. 

A significantly higher proportion of male versus female athletes did not meet the requirements for ALA (87.5% vs. 68.1%; *p* = 0.03), dietary fiber (70.8% vs. 27.7%; *p* ≤ 0.0001), and total water (70.8% vs. 44.7%; *p* = 0.01). A significantly higher portion of female in comparison to male athletes did not consume enough protein (70.2% vs. 25.0%; *p* ≤ 0.0001) and vitamin B_12_ (46.8% vs. 22.9%; *p* = 0.02).

## 4. Discussion

This is one of the first studies to comprehensively examine the adequacy of macronutrient and micronutrient intakes among endurance athletes and differences by sex. The present study showed that the majority of male and female endurance athletes are not meeting the requirements for several macronutrients and micronutrients with a few differences by sex.

Energy needs were not met by most athletes. This was unsurprising given that several previous studies have reported that many athletes do not meet their energy needs [10,11,12,13]. The majority of the athletes in the present study consumed less than the recommended amount of carbohydrates with no difference by sex. These results are corroborated by previous studies which have reported that many athletes, irrespective of sex did not meet the recommendations for carbohydrates [1,12,14,16,17]. Low carbohydrate intakes may lead to increased fatigue [47], negatively affect performance [48], and increase susceptibility to muscle damage [49] among athletes. Low carbohydrate intake may be especially detrimental to high-intensity exercise performance [50]. The present study also found that a significantly higher proportion of female athletes did not consume enough protein compared to male athletes. This is consistent with previous studies which found that female athletes were more likely to consume less than the recommended amount of protein versus male athletes [14,16]. Adequate protein intake is also essential for recovery and performance among athletes [50]. For instance, previous research has shown that low consumption of protein may decrease time to fatigue, which decreases performance and post-exercise recovery [51,52]. A significantly higher portion of our male compared to female athletes consumed less dietary fiber than recommended. This may be explained by a previous study which reported higher fruit intake among women [53]. Nevertheless, a previous study found a higher likelihood of low fiber intake among female compared to male athletes [16]. Low dietary fiber consumption may negatively impact gut microbiota composition and function [54], and this may cause increased inflammation and negatively affect athletic performance [54]. Moreover, when dietary fiber is fermented, short chain fatty acids are produced which increases mitochondrial function, energy production, and athletic performance [55].

None of the endurance athletes met the recommendations for EPA and DHA, and many did not have enough LA and ALA in their diet. Several studies have reported that a high proportion of athletes do not meet recommendations for these fatty acids [6,16]. Additionally, a significantly higher portion of our male compared to female athletes did not meet the recommendation for ALA. A possible explanation for differences in ALA by sex is that women engage in healthier eating practices compared to men [53]. Many of our participants also overconsumed dietary cholesterol, total fat, and saturated fat. Mielgo-Ayuso et al. [15] found that 64% of female volleyball players consume greater amounts of fat than recommended. Another study showed that male athletes consumed more than the recommended amount of cholesterol, fat, and saturated fat [1]. LA may decrease the uptake of lipids into adipocytes; therefore athletes who consume low amounts of linoleic acid might have difficulty maintaining a body weight conducive to optimal athletic performance [29,30]. Low consumption of ALA may lead to increased exercise-induced inflammatory markers (e.g., interleukin 6, tumor necrosis factor, and c-reactive protein), which may impact the health and performance of athletes [31]. Other fatty acids such as EPA and DHA have been shown to improve athletic performance by reducing soreness and enhance recovery by increasing the structural integrity of muscles following exercise [56]. EPA and DHA may also reduce the risk of injury or illness due to their anti-inflammatory effects [56]. Despite the benefits of fatty acids on athletic performance we found in our own lab that endurance athletes rarely use fatty acid supplementation [57] even though it has been shown to improve fatty acid profiles [58]. Further, high saturated fat intake may increase blood viscosity that could lead to poorer muscle oxidation and reduced athletic performance [59].

Many athletes consumed less than the recommended amount of several water-soluble vitamins including pantothenic acid, biotin, thiamine, vitamin B_12_, folate, and vitamin C. A number of previous studies have found that many athletes do not meet the recommendations for the aforementioned water-soluble vitamins [21,22,23,27,60]. In the present study, female athletes also had lower vitamin B_12_ intake and a significantly higher proportion of female athletes did not consume enough vitamin B_12_ compared to males. Janelle and Barr [28] have also reported a lower intake of vitamin B_12_ among female athletes, especially those who were vegetarian. Low intakes of pantothenic acid and biotin in athletes may influence an athlete’s ability to convert dietary protein, fat, and carbohydrates into energy during exercise, which may result in increased fatigue [61]. Athletes who do not consume enough vitamin C may have difficulty recovering from intense trainings and reduced performance because this vitamin plays an important role in collagen repair [25]. Chronic inadequate intake of thiamine may cause decreases in muscle tissue maintenance and repair and negatively impact performance [62]. Both vitamin B_12_ and folate play a role in erythropoiesis [25]. Athletes who do not consume enough of these nutrients are at increased risk of developing megaloblastic anemia which may result in symptoms such as fatigue and decreased endurance during exercise [25].

Numerous athletes did not meet the recommendations for the fat-soluble vitamins A, D, E, and K with no differences by sex. A number of studies have reported that these vitamins are under consumed by many male and female athletes [1,18,19,25,26]. However, these studies as well as the present study did not take into account the amount of vitamin D synthesized with exposure to sunlight. Low intake of vitamin D and calcium increases the risk for stress fractures among athletes [7,61]. Vitamin E, an antioxidant, plays a critical role in protecting the body from oxidative stress [63]. Athletes who do not consume enough vitamin E may experience increased muscle fatigue, muscle damage, and decreased immune function [61,63]. Low vitamin A intake may lead to increases in lactate levels resulting in decreased performance during strenuous exercise [64]. Moreover, athletes who consume lower than the recommended amount of vitamin K have been shown to have higher bone turnover and an increased risk for stress fractures [26].

Many athletes did not consume the recommended amount of total water (obtained through plain drinking water, beverages, and food) and minerals including potassium, choline, iron, magnesium, calcium, manganese, chromium, zinc, molybdenum, and selenium. Additionally, sodium intake was high among most participants. There were no significant differences by sex except for total water needs which were more likely to be met by females. This may be partly because of their greater intake of foods such as fruit that contain water [53]. Several studies have found that athletes do not meet the recommendations for total water, potassium, iron, magnesium, calcium, manganese, chromium, zinc, molybdenum, and selenium and consume too much sodium [9,16,20,21,24,27,61,65,66,67,68]. Water and electrolytes are needed for fluid balance and poor hydration can negatively affect training sessions and performance and lead to heat exhaustion, particularly in the southern regions of the U.S. where the majority of our study sample resided [61]. Consuming inadequate iron in athletes may lead to decreased oxygen transport, energy production, and aerobic capacity which could negatively impact athletic performance, increase lethargy, and fatigue [69,70]. Vegetarians may be even more susceptible to iron deficiency than non-vegetarians given their iron intake. Our study, however, found that no difference in the proportion of vegetarians (14.3%) and non-vegetarians (11.4%) who were not meeting their iron needs. Inadequate consumption of magnesium among athletes may decrease oxygen uptake during exercise, affect bone density, and increase the risk for stress fractures [71]. Athletes who do not consume adequate amounts of chromium and molybdenum may experience decreased glycogen stores, and increased fatigue during exercise [72,73]. Low zinc consumption among athletes may lead to deficits in muscle tissue repair, maintenance, growth, and energy production, and decreased immune functioning [61]. Athletes not consuming enough selenium or choline may have increased oxidative stress leading to poor recovery, fatigue, increased muscle soreness, and decreased performance [74,75].

The present study had several limitations. Our results may not be generalized to a wider population because most of the subjects were non-Hispanic White and educated. We used the 24 h dietary recall which may be subject to under-recall. To minimize this, dietary intake was assessed using the validated multiple-pass 24 h recall method [34] where participants were probed several times for the most commonly missed foods. Another limitation is that we did not have objective data such as serum nutrient, metabolomics concentrations, or use any clinical assessments in these athletes. This would limit our ability to assess certain nutrients such as vitamin D which is largely synthesized in the body [76]. Future studies should collect data on objective as well as subjective measures of nutrient intakes when assessing nutrient adequacy. The objective measures are seen as complementary rather than replacement for traditional validated dietary measures such as the one that we used in our study [65]. We did not get detailed information on supplement use. However, our aim was to examine if the athletes were meeting their nutrient requirements through their diet. A strength of the study is that nutrient adequacy was assessed using a comprehensive set of nutrients not previously evaluated among athletes. In addition, the present study had nearly an equal number of male and female endurance athletes.

For most athletes in the present study, it is likely that the number of calories consumed contributed to the nutrient inadequacies since most participants did not meet their energy needs. Previous studies have shown that athletes tend to make poor food choices which may also explain why many of our participants did not meet their nutrient needs [77,78]. The nutrient inadequacies may be addressed by consuming a diet that includes poultry without skin, fatty fish (e.g., salmon, trout, tuna, etc.), low-fat milk and dairy, whole grains (e.g., brown rice, whole-grain bread, whole-wheat pasta, etc.), green leafy vegetables (e.g., spinach, kale, collard greens, broccoli, etc.), orange and yellow fruits and vegetables (e.g., carrots, sweet potatoes, mangoes, papaya, etc.), nuts, seeds, and legumes in the appropriate amounts. To maintain adequate water intake, male and female athletes should drink 3.0 and 2.2 L/d, respectively [35], in the form of water, coffee and tea without sugar or cream, and some low-fat milk and 100% fruit juice. Athletes should also follow the American College of Sports Medicine guidelines for water and electrolyte intake before, during, and after exercise [61]. A multi-vitamin and mineral supplement is also recommended for athletes but not as a substitute for a healthy diet [61]. A nourishing diet is rich in phytochemicals as well as nutrients known to promote health [79].

## 5. Conclusions

In summary, the present study showed that many endurance athletes are not meeting the requirements for a number of macronutrients and micronutrients. There were few differences by sex, however. These results need to be confirmed with objective measures such as serum nutrient levels and metabolomics. Dietary counseling by a registered dietitian may be beneficial for athletes to achieve adequate nutrient intakes and for optimal performance, recovery, health, and prevention of injuries.

## 6. Contribution to the Field Statement

Many endurance athletes are not meeting their nutrient requirements. Therefore, may be necessary for athletes to consult with a registered dietitian to ensure that they are receiving the necessary nutrients for optimal health and performance. Dietitians and coaches should encourage athletes to consume a well-balanced diet that is rich in whole grains, green leafy vegetables, orange and red fruits and vegetables, lean meats, and low-fat milk and dairy. These findings need to be confirmed by a larger study. 

## Figures and Tables

**Table 1:** Participant characteristics of endurance athletes.

Variables	Total Sample (n = 95)	Female (n = 47)	Male (n = 48)	*p* *
Age (y)	34.9 ± 12.9	33.5 ± 11.0	36.2 ± 14.5	0.49
BMI (kg/m^2^)	23.6 ± 3.84	23.0 ± 2.81	24.2 ± 4.59	0.12
Race				
White	87 (91.6)	41 (87.2)	46 (95.8)	0.16
Other	8 (8.4)	6 (12.8)	2 (4.2)	
Ethnicity				
Hispanic	17 (16.8)	4 (8.6)	12 (25.0)	0.09
Non-Hispanic	79 (83.2)	43 (91.4)	36 (75.0)	
Education				
≤High school	2 (2.1)	0 (0.0)	2 (4.2)	0.51
Some college	25 (26.4)	10 (21.3)	15 (31.2)	
Bachelor’s degree	31 (32.6)	16 (34.0)	15 (31.3)	
Graduate degree	37 (38.9)	21 (44.7)	16 (33.3)	
Endurance Sport				
Cycling	18 (18.9)	6 (12.8)	12 (25.0)	0.27
Running	47 (49.5)	24 (51.1)	23 (47.9)	
Triathlon	23 (24.2)	14 (29.8)	9 (18.7)	
Swimming	2 (2.0)	0 (0.0)	2 (4.2)	
Rowing	5 (5.3)	3 (6.3)	2 (4.2)	
Smoking				
Yes	0 (0.0)	0 (0.0)	0 (0.0)	1
No	95 (100.0)	47 (100.0)	48 (100.0)	
Alcohol Consumption				
Yes	63 (66.3)	34 (72.3)	29 (60.4)	0.28
No	32 (33.7)	13 (27.7)	19 (39.6)	
Vegetarian				
Yes	7 (7.4)	6 (12.8)	1 (2.1)	0.06
No	88 (92.6)	41 (87.2)	47 (97.9)	
Chronic Condition				
Yes	61 (64.2)	30 (63.8)	31 (64.5)	1
No	34 (35.8)	17 (36.2)	17 (35.4)	
Medication Use				
Yes	35 (36.8)	19 (40.4)	16 (33.3)	0.51
No	60 (63.2)	28 (59.6)	32 (66.7)	
Supplement Use				
Yes	47 (49.5)	24 (51.1)	23 (47.9)	0.84
No	48 (50.5)	23 (48.9)	25 (52.1)	

Abbreviations: BMI, Body mass index. Categorical variables are presented as number and percent of subjects and continuous variables as mean ± standard deviation. * Fisher’s Exact test was used to compare the categorical variables and a Wilcoxon Rank-Sum test was used to compare the continuous variables between the female and male participants.

**Table 2:** Energy and dietary macronutrient intakes of female and male endurance athletes.

Nutrient	Total Sample (n = 95)	Female (n = 47)	Male (n = 48)	*p* *
Energy (kcal)	2283 (1655–2826)	1998 (1475–2441)	2539 (1996–3246)	**≤0.0001**
Protein (% energy)	18.9 (14.6–23.5)	18.6 (15.0–22.9)	18.9 (14.5–24.3)	0.88
Carbohydrate (% energy)	43.4 (33.7–57.1)	43.0 (34.8–56.3)	46.4 (33.0–62.2)	0.95
Total fat (% energy) ^⸹^	34.3 (58.5–42.7)	33.8 (28.7–44.3)	34.4 (28.3–41.0)	0.91
SFA (% energy)	10.1 (7.44–12.4)	9.53 (7.4–11.7)	10.2 (7.9–14.0)	0.36
PUFA (% energy)	4.3 (3.0–7.2)	4.4 (2.9–7.4)	4.1 (3.0–6.1)	0.60
MUFA (% energy)	8.4 (5.5–12.8)	8.2 (5.0–13.5)	8.7 (5.8–12.7)	0.82
LA (g)	7.53 (3.62–13.4)	7.29 (3.4–13.4)	7.8 (3.8–13.9)	0.84
ALA (g)	0.67 (0.38–1.34)	0.68 (0.39–1.4)	0.59 (0.37–1.3)	0.95
EPA (g)	0.00 (0.00–0.1)	0.00 (0.00–0.01)	0.00 (0.00–0.01)	0.84
DHA (g)	0.01 (0.00–0.06)	0.01 (0.00–0.04)	0.01 (0.00–0.07)	0.95
Cholesterol (mg)	286 (138–462)	217 (118–394)	369 (204–539)	**<0.01**
Dietary fiber (g)	27.5 (19.5–36.9)	28.2 (20.7–36.1)	27.3 (19.2–38.4)	0.86

Abbreviations: SFA, saturated fatty acids; PUFA, polyunsaturated fatty acids; MUFA, monounsaturated fatty acids; LA, linoleic acid; ALA, alpha-linoleic acid; EPA, eicosapentaenoic acid, DHA, docosahexaenoic acid. Energy and macronutrient values are presented as medians and 25th and 75th percentiles. ^⸹^ Total fat includes SFA, PUFA, MUFA, and other unspecified fat. * Wilcoxon Rank-Sum test was used to compare energy and dietary macronutrients by sex.

**Table 3:** Dietary micronutrient intakes of female and male endurance athletes.

Nutrient	Total Sample (n = 95)	Female (n = 47)	Male (n = 48)	*p* *
Vitamin A (μg)	543 (220–1065)	502 (201–744)	785 (364–1516)	0.051
Vitamin C (mg)	104 (39.9–202)	116 (60.1–197)	101 (38.8–205)	0.86
Vitamin D (μg)	2.0 (0.37–4.4)	1.6 (0.25–3.5)	2.0 (0.38–5.3)	0.37
Vitamin E (mg)	7.7 (4.2–13.9)	8.1 (4.3–14.6)	7.7 (3.8–13.4)	0.47
Vitamin K (μg)	90.1 (41.2–268)	90 (43.9–164)	91 (35.1–452)	0.57
Thiamine (mg)	1.2 (0.77–1.73)	1.0 (0.74–1.5)	1.3 (0.87–1.8)	0.13
Riboflavin (mg)	1.7 (1.2–2.3)	1.5 (1.2–2.2)	1.9 (1.2–2.9)	0.18
Niacin (mg)	26.6 (15.8–40.6)	22.4 (15.8–37.4)	29.2 (15.9–47.2)	0.12
Vitamin B_6_ (mg)	1.9 (1.29–2.77)	1.7 (1.2–2.5)	2.1 (1.4–2.9)	0.18
Folate (μg)	283 (138–470)	258 (131–399)	352 (142–530)	0.26
Vitamin B_12_ (μg)	2.8 (1.6–5.0)	2.0 (1.2–4.0)	3.9 (2.2–6.2)	**≤0.001**
Pantothenic acid (mg)	3.3 (2.1–5.7)	3.2 (2.1–5.2)	3.3 (1.9–6.0)	0.97
Biotin (μg)	7.9 (3.3–16.7)	7.9 (3.2–17.9)	7.9 (3.4–16.7)	0.82
Calcium (mg)	888 (630–1272)	911 (551–1298)	833 (637–1266)	0.95
Copper (mg)	1.1 (0.72–1.6)	1.1 (0.62–1.8)	1.1 (0.76–1.4)	0.74
Manganese (mg)	1.7 (0.86–2.6)	1.6 (0.74–2.6)	1.9 (0.93–2.7)	0.52
Iron (mg)	15.0 (10.4–19.9)	11.9 (10.1–17.2)	17.2 (12.3–21.2)	**<0.01**
Magnesium (mg)	267 (192–387)	281 (180–402)	259 (203–367)	0.86
Phosphorus (mg)	1027 (712–1443)	968 (697–1278)	1069 (732–1780)	0.13
Selenium (μg)	78.5 (38.7–115.2)	68.4 (35.5–100)	95.1 (44.7–143)	**0.02**
Chromium (μg)	1.69 (0.33–5.5)	1.7 (0.26–5.2)	1.9 (0.62–5.5)	0.72
Molybdenum (μg)	6.9 (.48–16.6)	7.2 (2.3–15.0)	5.7 (0.00–20.5)	0.76
Choline (mg)	238 (122–389)	200 (109–332)	287 (147–509)	**0.03**
Zinc (mg)	8.4 (5.7–12.0)	7.1 (5.4–9.4)	9.7 (5.8–14.1)	**0.03**
Potassium (mg)	2650 (1596–3657)	2360 (1442–3490)	3143 (1919–3945)	0.10
Sodium (mg)	3835 (2785–4658)	3776 (2451–4463)	3932 (2935–5422)	0.06
β-carotene (μg)	1706 (421–6906)	1706 (874–4136)	1805 (210–9799)	0.97
Total water (L)	2.90 (2.14–4.07)	2.79 (2.14–3.98)	3.00 (2.15–4.17)	0.99

Micronutrient values are presented as medians and 25th and 75th percentiles. * Wilcoxon Rank-Sum test was used to compare micronutrient intakes by sex.

**Table 4:** Proportion of endurance athletes with inadequate nutrient intakes.

	% Who Did Not Meet Requirement			
Nutrient Requirement/d	Total Sample (n = 95)	Female (n = 47)	Male (n = 48)	*p* *
Energy ^€^ (kcal)	76.8	76.6	77.1	1.0
Protein ^†^ (M/F: 1.5 g/kg)	47.4	70.2	25.0	**≤0.0001**
Carbohydrate ^†^ (M/F: 8 g/kg)	95.8	97.9	93.8	0.62
Total fat^†^ (M/F: 20–35% energy)	47.4	46.8	47.9	1.0
Saturated fat ^⁋^ (M/F: <10% of total energy)	50.5	48.9	52.1	0.84
Linoleic acid ^ǁ^ (M: 17 g (19–50 y); 14g (≥51 y)) (F: 12 g (19–50 y); 11g (≥51 y))	75.8	68.1	83.3	0.10
alpha-linolenic acid ^ǁ^ (M: 1.6 g; F: 1.1 g)	77.9	68.1	87.5	**0.03**
EPA+DHA ^§^ (M/F: 0.5 g)	96.8	97.9	95.8	1.0
Dietary cholesterol ^§^ (M/F < 300 mg)	48.4	40.4	56.3	0.15
Dietary fiber ^ǁ^ (M: 38 g (19–50 y); 30 g (≥51 y)) (F: 25 g (19–50 y); 21 g (≥51 y))	49.5	27.7	70.8	**≤0.0001**
Vitamin A ^⸸⸹^ (M: 625 µg; F: 500 µg)	46.3	48.9	43.8	0.68
Vitamin C ^⸸^ (M: 75 mg; F: 60 mg)	30.5	23.4	37.5	0.18
Vitamin D ^⸸^ (M/F: 10 µg)	93.7	95.7	91.7	0.68
Vitamin E ^⸸⸹^ (M/F: 12 mg)	71.6	68.1	75.0	0.50
Vitamin K ^ǁ^ (M: 120 µg; F: 90 µg)	54.7	51.1	58.3	0.54
Thiamine ^⸸^ (M: 1.0 mg; F: 0.9 mg)	40.0	42.6	37.5	0.68
Riboflavin ^⸸^ (M: 1.1 mg; F: 0.9 mg)	18.9	14.9	22.9	0.43
Niacin ^⸸⸹^ (M: 12 mg; F: 11 mg)	9.5	10.6	8.3	0.74
Vitamin B_6_^⸸^ (M: 1.1 mg (19–50 y); 1.4 mg (≥51 y)) (F: 1.1 mg (19–50 y); 1.3 mg (≥51 y))	21.1	21.3	20.8	1.0
Folate ^⸸⸹^ (M/F: 320 µg)	54.7	61.7	47.9	0.22
Vitamin B_12_^⸸^ (M/F: 2 µg/d)	34.7	46.8	22.9	**0.02**
Pantothenic acid ^ǁ^ (M/F: 5 mg)	70.5	74.5	66.7	0.50
Biotin ^ǁ^ (M/F: 30 µg)	83.2	83.0	83.3	1.0
Calcium ^⸸^ (M: 800 mg (19–70 y); 1000 mg (>70 y)) (F: 800 mg (19–50 y); 1000 mg (≥51 y))	42.1	42.6	41.7	1.0
Copper ^⸸^ (M/F: 700 µg)	24.2	25.5	22.9	0.81
Manganese ^ǁ^ (M: 2.3 mg; F: 1.8 mg)	58.9	59.6	58.3	1.0
Iron ^⸸^ (M: 6 mg) (F: 8.1 mg (19–50); 5 mg (≥51 y))	11.6	14.9	8.3	0.36
Magnesium ^⸸^ (M: 330 mg (19–30 y); 350 mg (≥31 y)) (F: 255 mg (19–30 y); 265 mg (≥31 y))	56.8	46.8	66.7	0.06
Phosphorus ^⸸^ (M/F: 580 mg)	18.9	21.3	16.7	0.61
Selenium ^⸸^ (M/F: 45 µg)	32.6	38.3	27.1	0.28
Chromium ^ǁ^ (M: 35 µg (19–50 y); 30 µg (≥51 y)) (F: 25 µg (19–50 y); 20 µg (≥51 y))	91.6	89.4	93.8	0.49
Molybdenum ^⸸^ (M/F: 34 µg)	93.7	95.7	91.7	0.68
Choline ^ǁ^ (M: 550 mg; F: 425 mg)	85.3	89.4	81.3	0.23
Zinc ^⸸^ (M: 9.4 mg; F: 6.8 mg)	46.3	46.8	45.8	1.0
Potassium ^ǁ^ (M: 3400 mg; F: 2600 mg)	56.8	55.3	58.3	0.84
Sodium ^ǁ^ (M/F: <1500 mg)	94.7	91.5	97.9	0.20
Total water ^ǁ^ (M: 3.7 L/d; F: 2.7 L/d)	57.9	44.7	70.8	**0.01**

Abbreviations: M, male; F, female; EPA, eicosatetraenoic; DHA, docosahexaenoic acid. * Fisher’s Exact test was used to compare the portion of male and female endurance athletes that did not meet the requirements for macro- and micro-nutrient intakes. ^€^ Estimated energy requirement [46] ^†^ American College of Sports Medicine guidelines [36]. ^⁋^ 2020–2025 Dietary Guidelines for Americans guidelines [37]. ^§^ American Heart Association guidelines [38]. ^ǁ^ Adequate Intakes [35,40,42,44]. ^⸸^ Estimate Average Requirements [39,40,42,43,44]. ^⸹^ vitamin A; retinol activity equivalents, vitamin E; alpha-tocopherol, niacin; niacin equivalents, folate; dietary folate equivalents.

## Data Availability

The data presented in this study are available upon reasonable request from the corresponding author.
